# Electrospray Ionization Mass Spectrometry of Transferrin: Use of Quadrupole Mass Analyzers for Congenital Disorders of Glycosylation

**DOI:** 10.5702/massspectrometry.A0103

**Published:** 2022-04-15

**Authors:** Yoshinao Wada, Nobuhiko Okamoto

**Affiliations:** 1 Department of Obstetric Medicine, Osaka Women’s and Children’s Hospital (OWCH), 840 Murodo-cho, Izumi, Osaka 594–1101, Japan; 2 Department of Molecular Medicine, Osaka Women’s and Children’s Hospital (OWCH), 840 Murodo-cho, Izumi, Osaka 594–1101, Japan; 3 Department of Medical Genetics, Osaka Women’s and Children’s Hospital (OWCH), 840 Murodo-cho, Izumi, Osaka 594–1101, Japan

**Keywords:** congenital disorder of glycosylation, ESI, *N*-glycosylation, transferrin, quadrupole mass spectrometer

## Abstract

Electrospray ionization (ESI) mass spectrometry of transferrin can be used to diagnose congenital disorders of glycosylation (CDG) by detecting abnormal *N*-glycosylation due to reduced site occupancy or processing failure. Time-of-flight mass spectrometers are widely used to separate 25–45 charged ions in the *m*/*z* 1,700–3,000 range, and a summed zero-charge mass distribution is generated despite the risk of improper deconvolution. In this study, the low *m*/*z* region of the multiply-charged ion mass spectrum enabled a robust analysis of CDG. A triple quadrupole mass spectrometer, the standard instrument for newborn screening for inborn errors of metabolism, permitted the identification of the key ions characteristic of different types of CDG affecting *PMM2*, *ALG14*, *SLC35A1*, *SLC35A2*, *MAN1B1* and *PGM1* in the *m*/*z* 1,970–2,000 region. Charge deconvolution was used as a complementary tool for validating the findings. It was necessary to set a cutoff level for the evaluation, since small peaks indicating glycosylation failure or reduced sialylation were observed, even in control subjects. This method and workflow facilitates the implementation of MS-based analyses and the screening of CDG in clinical laboratories.

## INTRODUCTION

Congenital disorders of glycosylation (CDG), an expanding group of genetic diseases related to the glycosylation of proteins and lipids, are caused by mutations in over 140 genes.^1)^ CDG are diagnosed by genetic and glycoprotein analysis of the patients with non-specific clinical conditions such as epilepsy and developmental delay.^2)^ Transferrin, an 80 kDa glycoprotein, is the best analyte for detecting abnormal *N*-glycosylation due to its low level of microheterogeneity. It contains two *N*-glycosylation sites that are occupied by biantennary oligosaccharides with terminal *N*-acetylneuraminic acid (NeuAc, or sialic acid) units. Fully sialylated transferrin or tetrasialo-transferrin is the major (>90%) isoform in the serum of control subjects. Historically, abnormal glycosylation in CDG was classified into two types, *i.e.*, CDG-I and II, based on isoelectric focusing (IEF) patterns. A lack of a glycan at one or two glycosylation sites produces disialo- or asialo-transferrin, which is referred to as CDG-I. Immature or partially truncated glycans that form CDG-II are characterized by the presence of trisialo- and monosialo-transferrin in IEF. This classification is still in use, because these types represent defective stages in the *N*-glycosylation pathway; CDG-I is derived from a defect in the formation of glycans on a membrane-anchored lipid dolichol or the subsequent *en bloc* transfer of the dolichol-linked glycans to nascent proteins in the endoplasmic reticulum (ER), and CDG-II occurs in the latter process of glycan maturation in the ER and the Golgi apparatus.

Mass spectrometry (MS) has a distinct advantage over IEF since its first application to CDG in 1992,^3)^ when the electrospray ionization (ESI) MS of transferrin from CDG-I patients demonstrated that the “disialo-transferrin” was a molecule lacking one entire glycan but not a form that contained truncated glycans. Subsequently, it was found that MS could be used to characterize an alteration in the glycoprofile,^4)^ namely the relative abundance of each glycoform, characteristic of CDG-II. With these excellent capabilities, MS has now become the key tool for diagnosing CDG.^5,6)^

ESI of transferrin generates multiply-charged ions bearing 25–45 protons, therefore requiring a wide mass range for mass separation up to *m*/*z* 3,000. For mass range and sensitivity reasons, a quadrupole time-of-flight (QTOF) type of mass separator is widely used for CDG.^7)^ On the other hand, there are few reports of the use of quadrupole (Q) type mass analyzers for this purpose.^8,9)^ Triple Q type mass spectrometers are the most popular MS instruments in clinical laboratories. While they are routinely employed in newborn screening for inborn errors of metabolism (IEMs), the mass scan range is often limited up to *m*/*z* 2,000. In this study, transferrin samples from CDG patients were analyzed by ESI-Q mass spectrometry. The limited mass range was not a critical weakness for the diagnosis of CDG, and a narrow mass range of less than *m*/*z* 2,000 showed sufficient capacity to permit different types of CDG to be identified.

## MATERIALS AND METHODS

### Patients and ethical approval

Sera without personally identifiable information were obtained from the doctors in charge of the patients at the Osaka Women’s and Children’s Hospial (OWCH) for the diagnosis of CDG. The patients were affected by multisystem diseases of unknown etiology. A genetic diagnosis was made before or after the molecular abnormality was identified by MS. This study has been approved by the institutional review board of OWCH.

### Immunopurification of transferrin

Immunopurification was performed according to a previously reported method.^10)^ Briefly, an affinity column was prepared using a rabbit (DAKO, Glostrup, Denmark) or a goat (Invitrogen, Thermo-Fisher Scientific, Waltham, MA, USA) polyclonal antibody against human transferrin and a ligand-coupling Sepharose column (HiTrap NHS-activated HP, GE Healthcare, Piscataway, NJ, USA), and the antibody-coupled Sepharose was then recovered from the column. A 10-μL portion of serum was mixed with a 20-μL slurry of the antibody-coupled Sepharose in 0.5 mL of phosphate-buffered saline (PBS), and the solution was incubated at 4°C for 30 min. After washing with PBS, the transferrin was eluted from the Sepharose with 0.1 M glycine–HCl buffer at pH 2.5.

### Mass spectrometry

Liquid chromatography MS was carried out by an API4500 ESI-triple Q mass spectrometer (Sciex, Framingham, MA, USA) or a QSTAR ESI-QTOF mass spectrometer (Sciex) connected to a C4 reversed phase column (2 mm diameter and 10 mm length, GL Sciences, Tokyo, Japan). After sample injection, the column was washed with 0.1% formic acid at a flow rate of 0.2 mL/min, and then eluted with 60% acetonitrile/0.1% formic acid at a flow rate of 0.05 mL/min.

API4500 was operated in the positive Q1 MS mode with the optimized parameters as follows: gas temperature was at 150°C, curtain gas pressure was 10 psi, ion source gas pressure was 16 psi, IonSpray voltage was 5.5 kV, declustering potential was 150 V, and entrance potential was 10 V. The full scan range was set from 1,780 to 2,000, and the scan rate was 10 Da/s. Polypropylene glycol was used for mass calibration.

QSTAR was operated in the positive TOF MS mode. The optimized parameters were as follows: gas temperature was ambient, nebulizer gas (GS1) was 40 psi, curtain gas pressure was 50 psi, IonSpray voltage was 5.5 kV, and declustering potential was 100 V. The full scan range was set from 1,500 to 3,000. The pulser frequency was 5 kHz and pulse duration was 20 μs. Sodium trifluoroacetate was used for mass calibration.^11)^

The zero-charge mass spectrum was generated by the Promass protein deconvolution software described above.

### Statistical analysis

Statistical analysis was performed by using JMP statistical analysis software (SAS Institute, NC, USA).

## RESULTS AND DISCUSSION

### Molecular mass of transferrin and isotopic distribution

**Figure 1:**
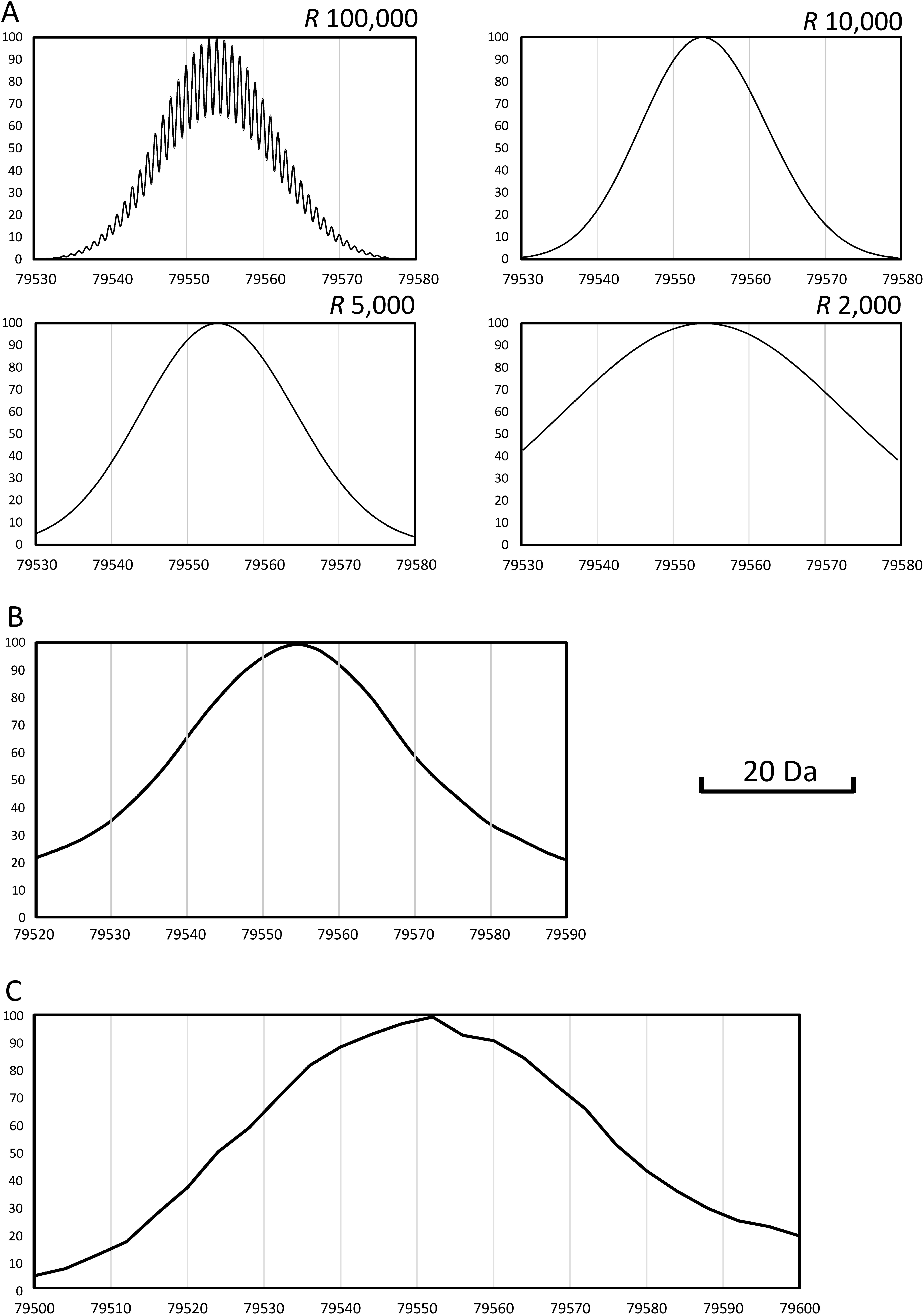
Fig. 1. Peak profile of transferrin. (A) Theoretical isotope distribution at different resolutions, as calculated by enviPat. (B) Charge-deconvoluted [M+40H]^40+^ ions measured by a QTOF mass spectrometer. (C) Charge-deconvoluted [M+40H]^40+^ ions measured by a Q mass spectrometer. Deconvolution was manually performed. Horizontal scale is the same for all figures.

**Figure 2:**
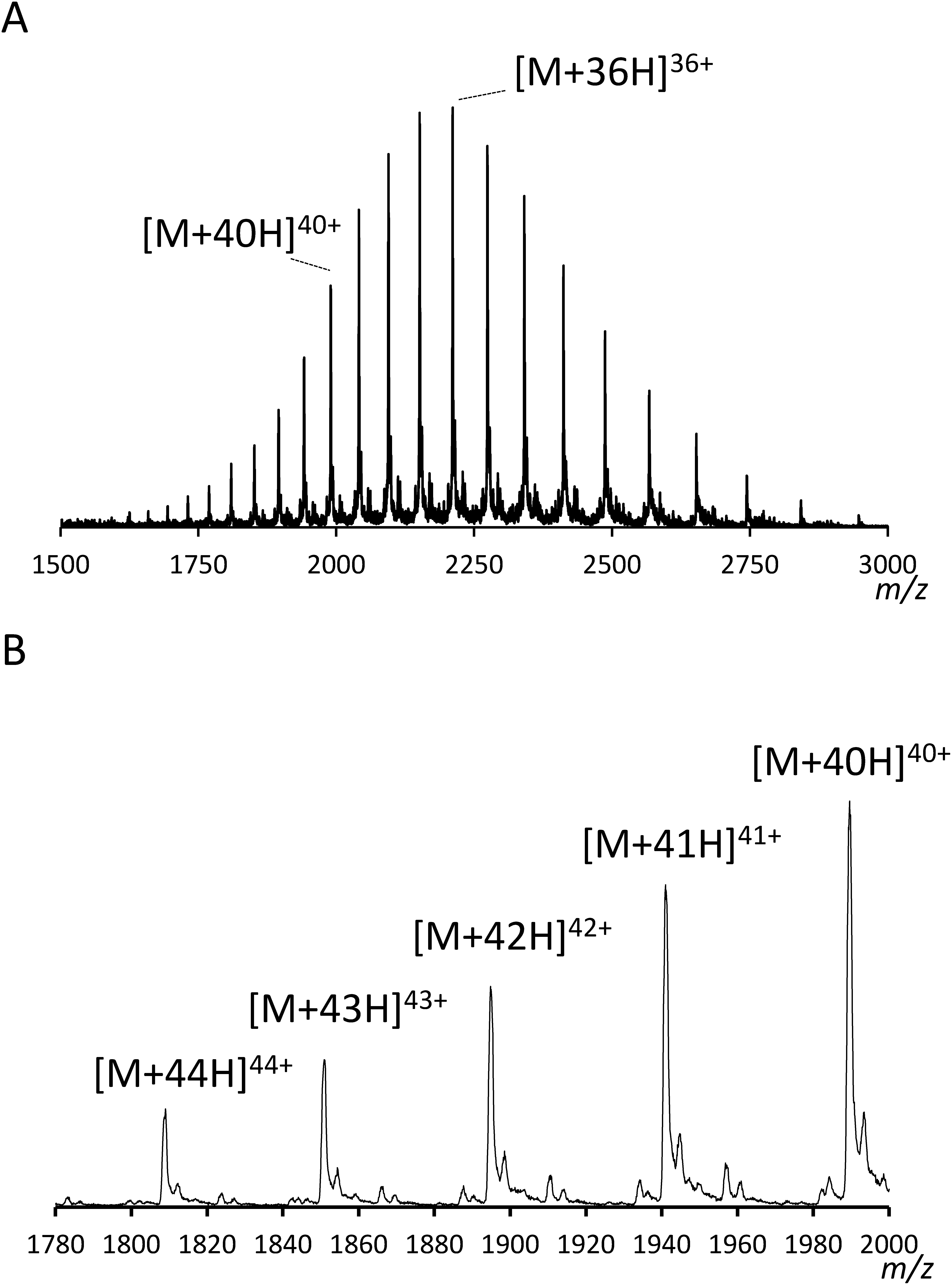
Fig. 2. ESI mass spectrum of transferrin. (A) QTOF mass spectrum. (B) Q mass spectrum.

Human serotransferrin (UniProt accession number P02787) is composed of 679 amino acid residues. The molecular mass (average mass) of the unglycosylated transferrin is 75,156.9 (C3306 H5096 N912 O1002 S47), when 19 disulfide bonds are present. In the UniProt record, the sequence of a predominant electrophoretic variant, C1 or TF*C1, contains isoleucine at residue 448. However, according to the public-domain archive for human single nucleotide variations, dbSNP (rs2692696), the residue is valine in the Japanese and other ethnic groups in the world, and therefore the molecular mass is 75,142.9 (C3305 H5094 N912 O1002 S47). The major (>90%) glycoform of serum transferrin is disialylated biantennary oligosaccharide (C84 H136 N6 O61; 2,206.0 for average mass), and it is partially fucosylated.^12)^ In addition, a small fraction of transferrin has a triantennary oligosaccharide. The molecular mass of serum transferrin with two disialylated biantennary glycans, or tetrasialotransferrin, is 79,554.9 (C3473 H5366 N924 O1124 S47). The theoretical isotope pattern, as calculated by enviPat Web 2.4 (https://www.envipat.eawag.ch/)^13)^ at different resolutions is shown in Fig. 1A. Distribution of the isotopic cluster is wide, and the peak width at a half maximum is approximately 15, 20, 25, and 45 Da at resolutions of 100,000, 10,000, 5,000, and 2,000, respectively. The ESI mass spectrum of a sample of transferrin obtained from a control subject was acquired by a QTOF mass spectrometer with a resolving power of approximately 5,000 (Fig. 2A), and the [M+40H]^40+^ ion was charge-deconvoluted (Fig. 1B). The peak width was 38 Da, which was larger than the theoretical value of 25 Da. The ESI-Q mass spectrum was then acquired by an instrument whose mass range was limited to *m*/*z* 2,000 for specific use in IEMs screening (Fig. 2B). The resolution was approximately 2,000 at *m*/*z* 1,800–2,000. The peak width of the charge-deconvoluted [M+40H]^40+^ ion of the ESI-Q mass spectrum was 52 Da (Fig. 1C). The peak broadening, observed in both QTOF-MS and Q-MS, would be expected to depress the accuracy owing to ambiguous reading of the peak centroid or the overlapping of unknown peaks adjacent to the target.

https://doi.org/10.50893/data.massspectrometry.19446803

### Key glycoforms of CDG

**Figure 3:**
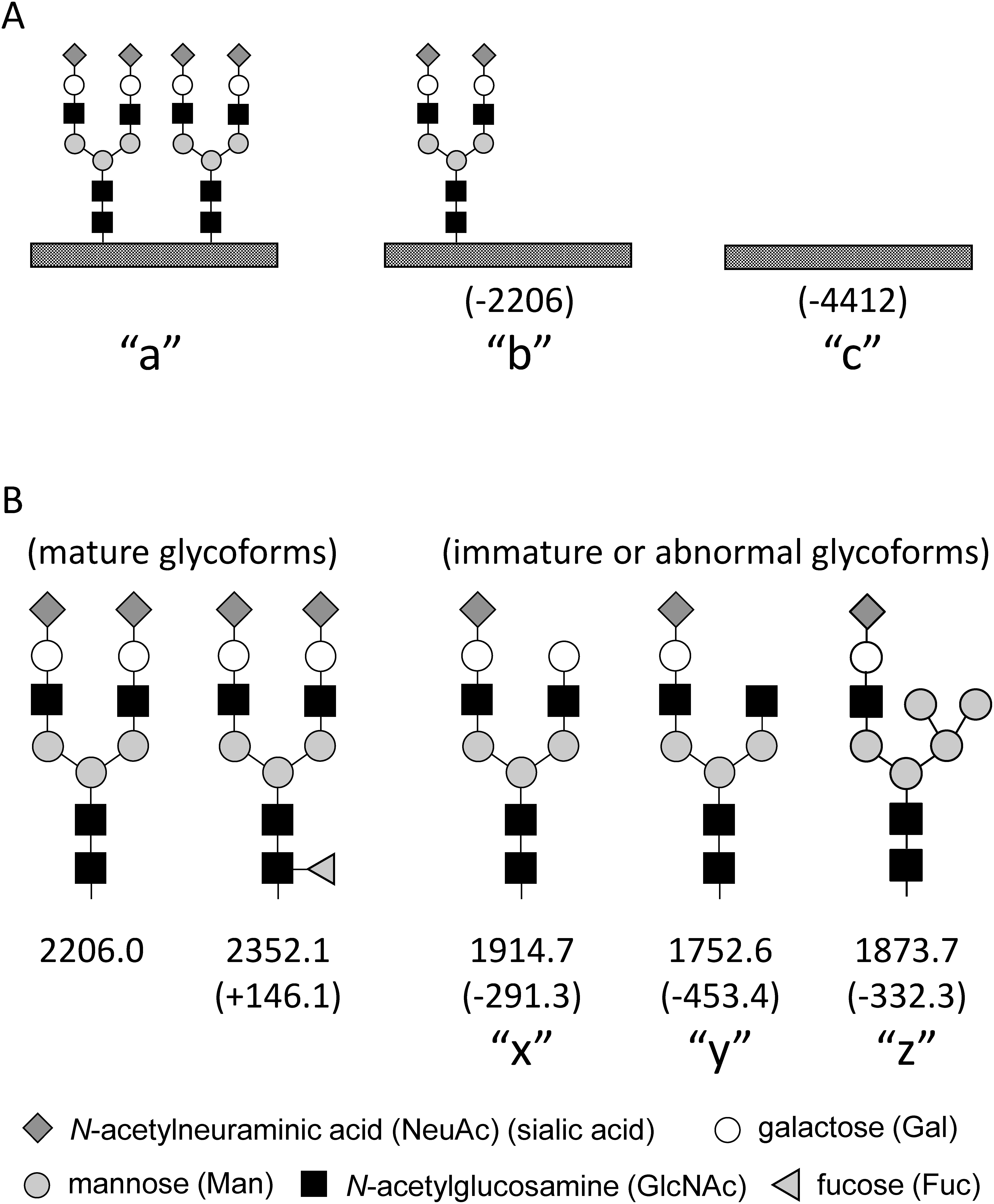
Fig. 3. Aberrant molecules and glycoforms of transferrin in CDG. Difference in average mass from the normal structure is indicated in parentheses. (A) Normal (a) and CDG-I type aberrant molecules (b, c). (B) Normal and aberrant glycoforms (x, y, z). Residue mass (average mass) is indicated below each glycoform.

**Figure 4:**
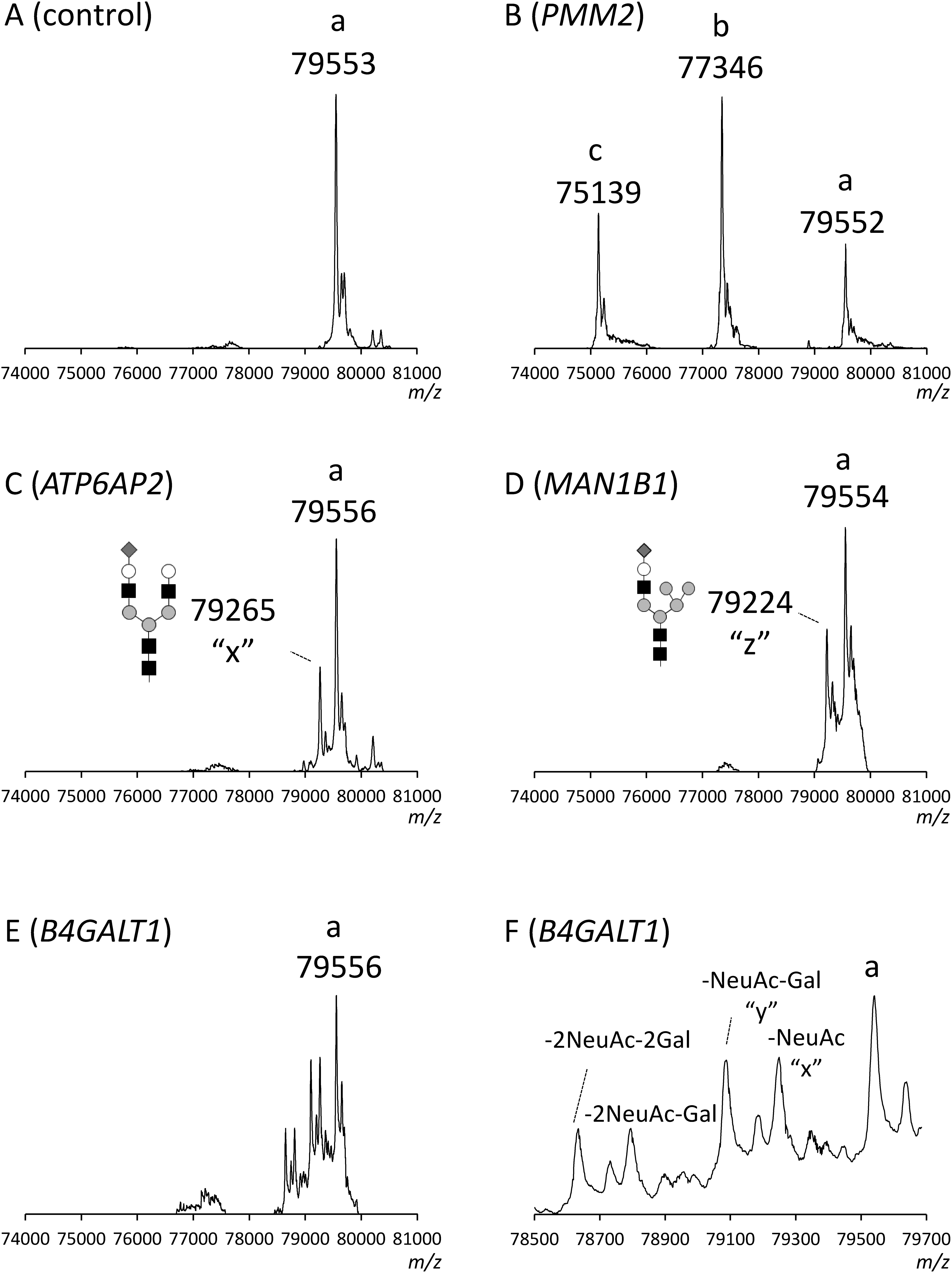
Fig. 4. Charge-deconvoluted spectra of various types of CDG. The ESI-QTOF mass spectrum was transformed by Promass protein deconvolution software (Thermo-Fisher Scientific). Aberrant molecules with decreased site occupancy (b, c) and immature or abnormal glycoforms (x, y, z) are indicated. Mass range 78,500–79,700 of E is depicted in F. PMM2-CDG is CDG-I, and others are CDG-II.

The major molecular or glycoform phenotypes of transferrin observed in CDG are illustrated with their masses in Fig. 3. It should be noted here that the mature/normal glycoform, disialylated biantennary oligosaccharide, is present in CDG and is the predominant form with the exception of some CDG-I type diseases. Since glycosylation is a post-translational modification, the core protein sequence is the same for normal and abnormal species in each patient. This helps in characterizing aberrant glycoforms based on the size of the mass shift from normal species. As shown in Fig. 3, the mass difference between the mature glycoform(s) and other forms exceeds 100 Da, suggesting that ESI-MS, either by QTOF or Q, can detect a transferrin molecule bearing abnormal glycoforms. Indeed, they were separated from each other in the ESI-QTOF mass spectra shown in Fig. 4.

### Charge-deconvoluted spectrum *vs.* multiply-charged ion mass spectrum

**Figure 5:**
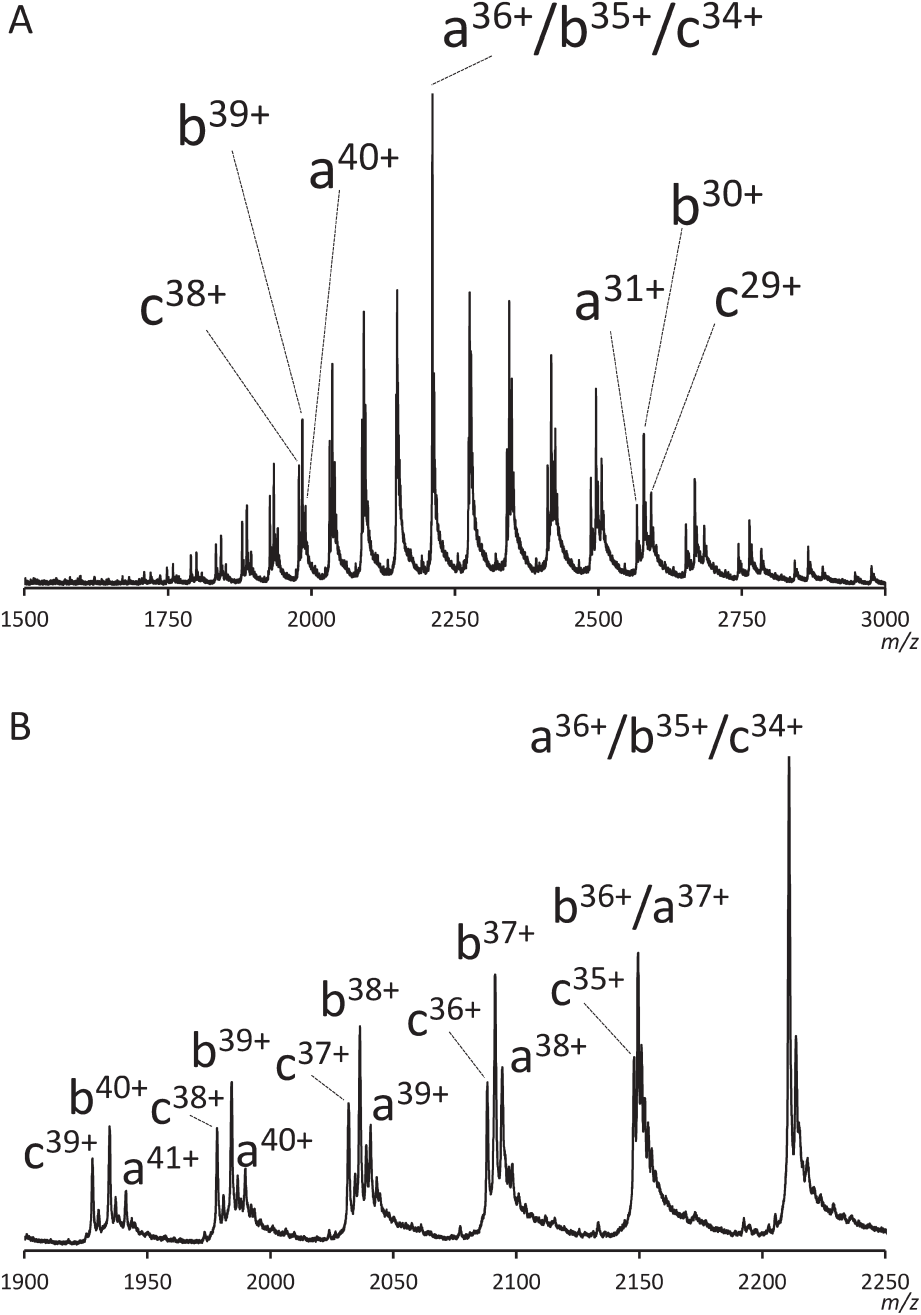
Fig. 5. ESI-QTOF mass spectrum of PMM2-CDG. (A) The multiply-charged ions of three transferrin isoforms are completely overlapped at *m*/*z* 2,211. See the supplementary Fig. 1S for the magnified mass spectrum. (B) Three isoforms are separated from each other below *m*/*z* 2,100.

**Figure 6:**
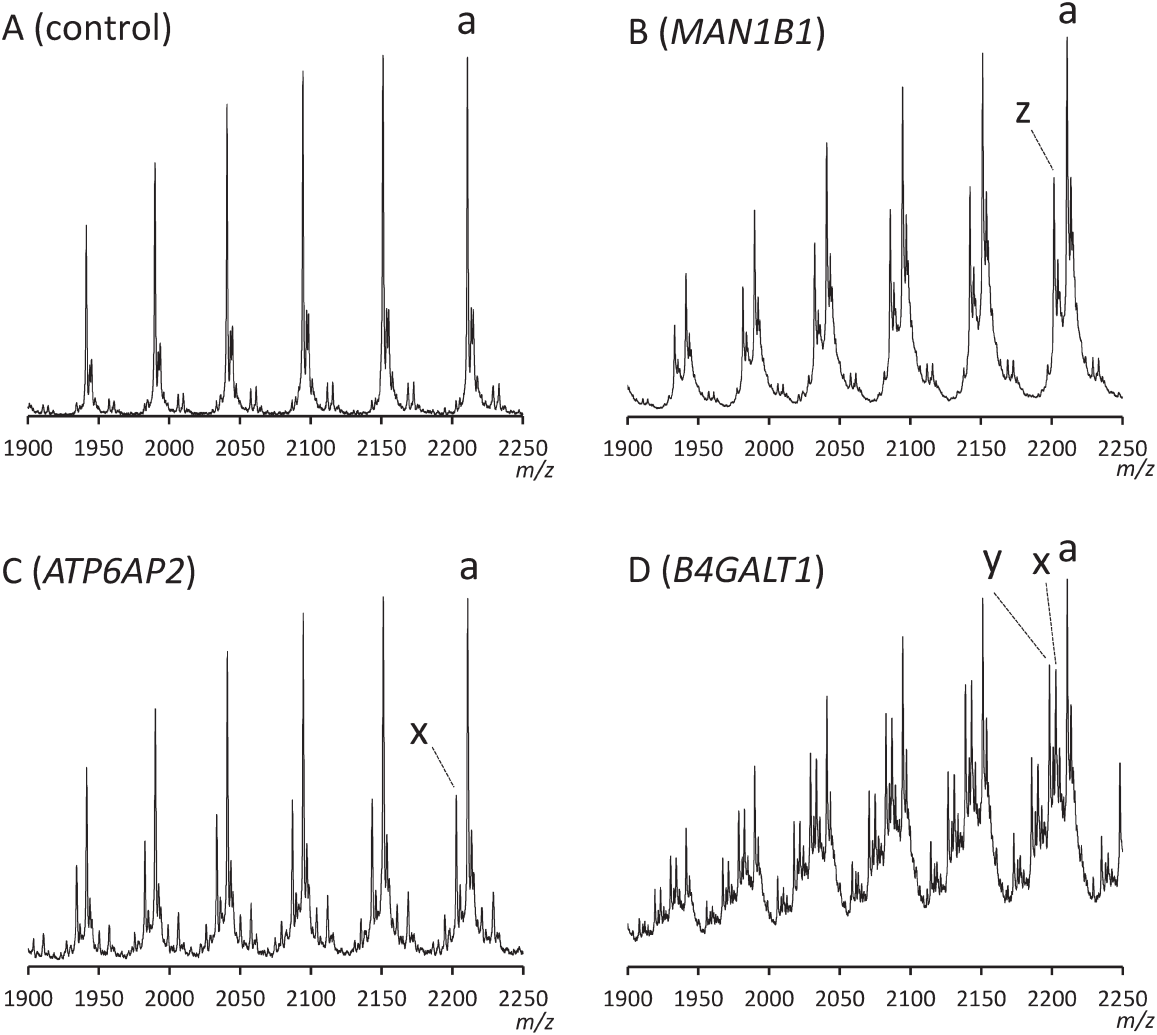
Fig. 6. ESI-QTOF mass spectrum of various CDG-II types. Immature or abnormal glycoforms (x, y, z) are indicated.

The deconvolution program is a tool that allows the ESI mass spectrum of multiply-charged ions to be transformed into a summed zero-charge mass distribution. Deconvolution aids in interpretation but a proper parameter setting is required to generate a valid spectrum, thus risking pitfalls.^10)^ For example, a small but diagnostic peak of CDG-I disappeared after a small change in the parameters (Supplementary Figure S2). Furthermore, in the ESI mass spectrum of PMM2-CDG, the *m*/*z* values of [M+36H]^36+^ ions of normal transferrin, [M+35H]^35+^ ions lacking one glycan, and [M+34H]^34+^ ions lacking two glycans were found to be 2,210.9, 2,211.0, and 2,211.1, respectively, and are completely overlapped (Figs. 5A and 1S). Similarly, the peaks for [M+33H]^33+^ ions lacking one glycan at *m*/*z* 2,344.9 and for [M+34H]^34+^ ions of fucosylated normal transferrin at *m*/*z* 2,345.1 were very close (Fig. 1S). Dealing with these overlapping ions would be challenging. On the other hand, the original multiply-charged ion mass spectrum in the lower mass region was free from overlapping, and the key glycoforms characterizing various types of CDG were detectable in a range below *m*/*z* 2,100 (Figs. 5B and 6).

https://doi.org/10.50893/data.massspectrometry.19446827

https://doi.org/10.50893/data.massspectrometry.19446833

### Analysis by ESI-Q MS up to *m*/*z* 2,000

**Figure 7:**
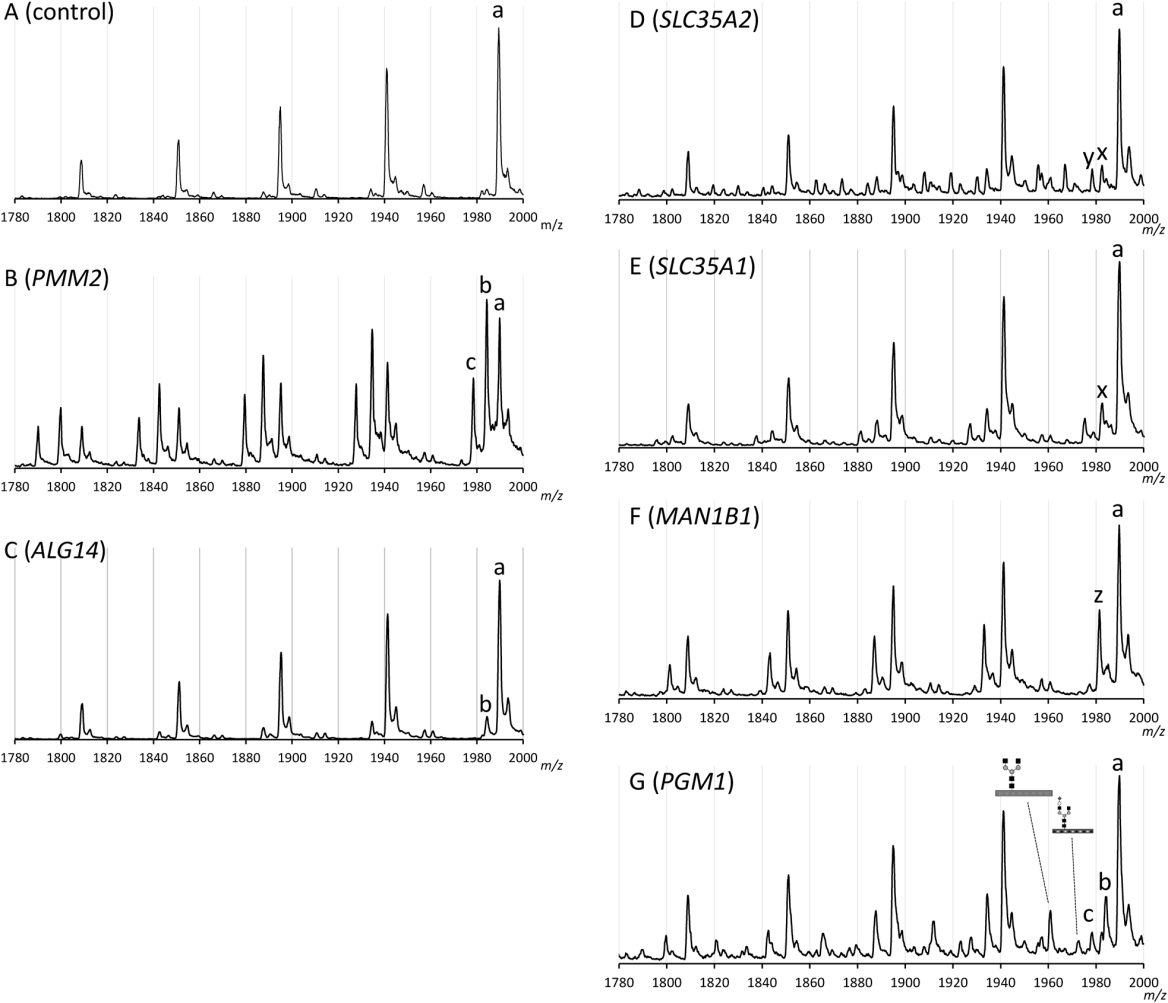
Fig. 7. ESI-Q mass spectrum of transferrin in various types of CDG. PMM2- and ALG14-CDG are CDG-I, and SLC35A2-, SLC35A1-, and MAN1B1 are CDG-II. PGM1-CDG shows the mixed CDG-I and II pattern.

**Figure 8:**
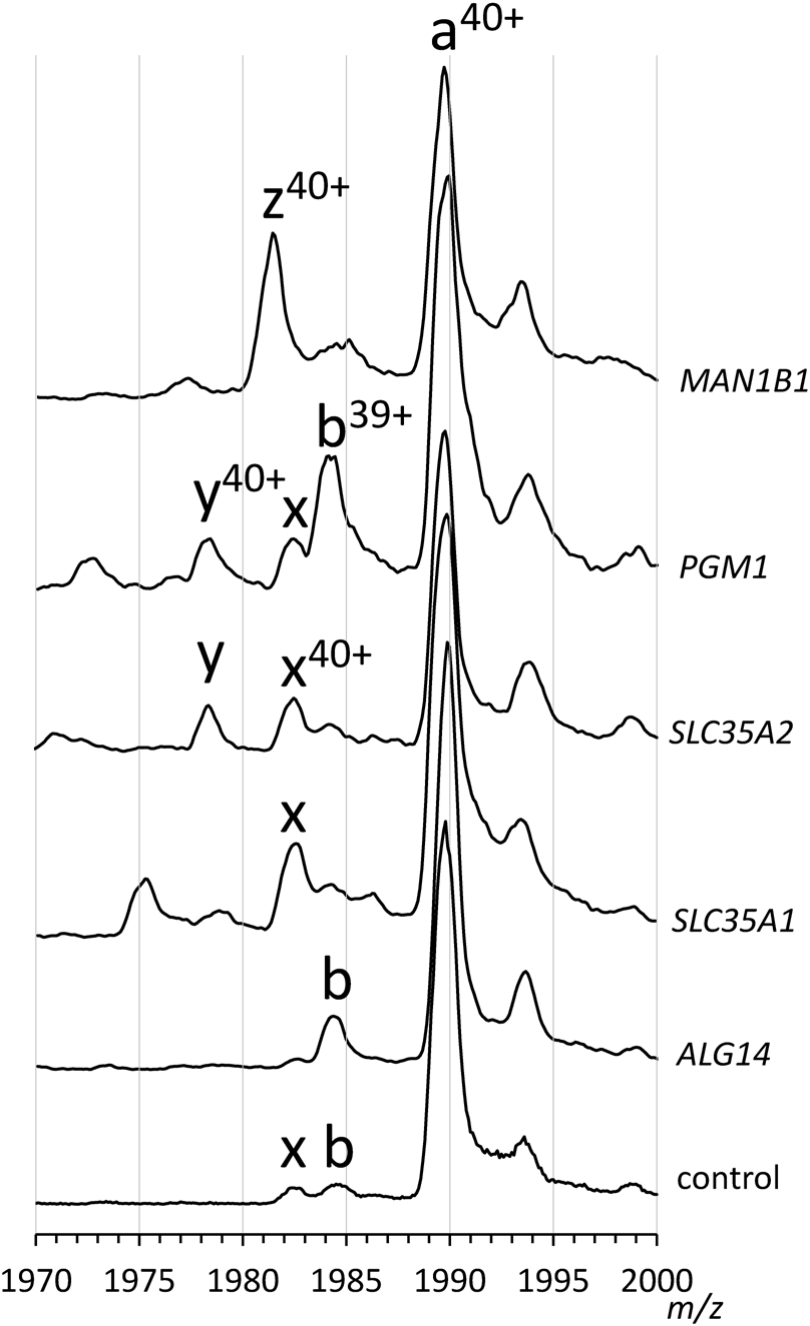
Fig. 8. Enlarged ESI-Q mass spectrum. The selected mass range *m*/*z* 1,970–2,000 in Fig. 7 is shown.

These findings suggest that a reliable analysis is possible even with a mass spectrometer with a limited mass range. Various types of CDG were analyzed by a Q mass analyzer whose mass range was limited up to *m*/*z* 2,000 (Fig. 7). Key molecules characterizing CDG were identified, and the forms characterized as CDG-I and CDG-II type diseases were discriminated against each other in a mass window from *m*/*z* 1,970 to *m*/*z* 2,000. As shown in Fig. 8, the peaks at *m*/*z* 1,984.3 and *m*/*z* 1,982.6 were 39-charged “b” and 40-charged “x,” respectively. Form “b” lacks one glycan, indicating reduced site occupancy characteristics of CDG-I. Form “x” lacks one sialic acid and is the common molecular phenotype in many CDG-II diseases.

https://doi.org/10.50893/data.massspectrometry.19446836

### Statistical evaluation for diagnosis

**Figure 9:**
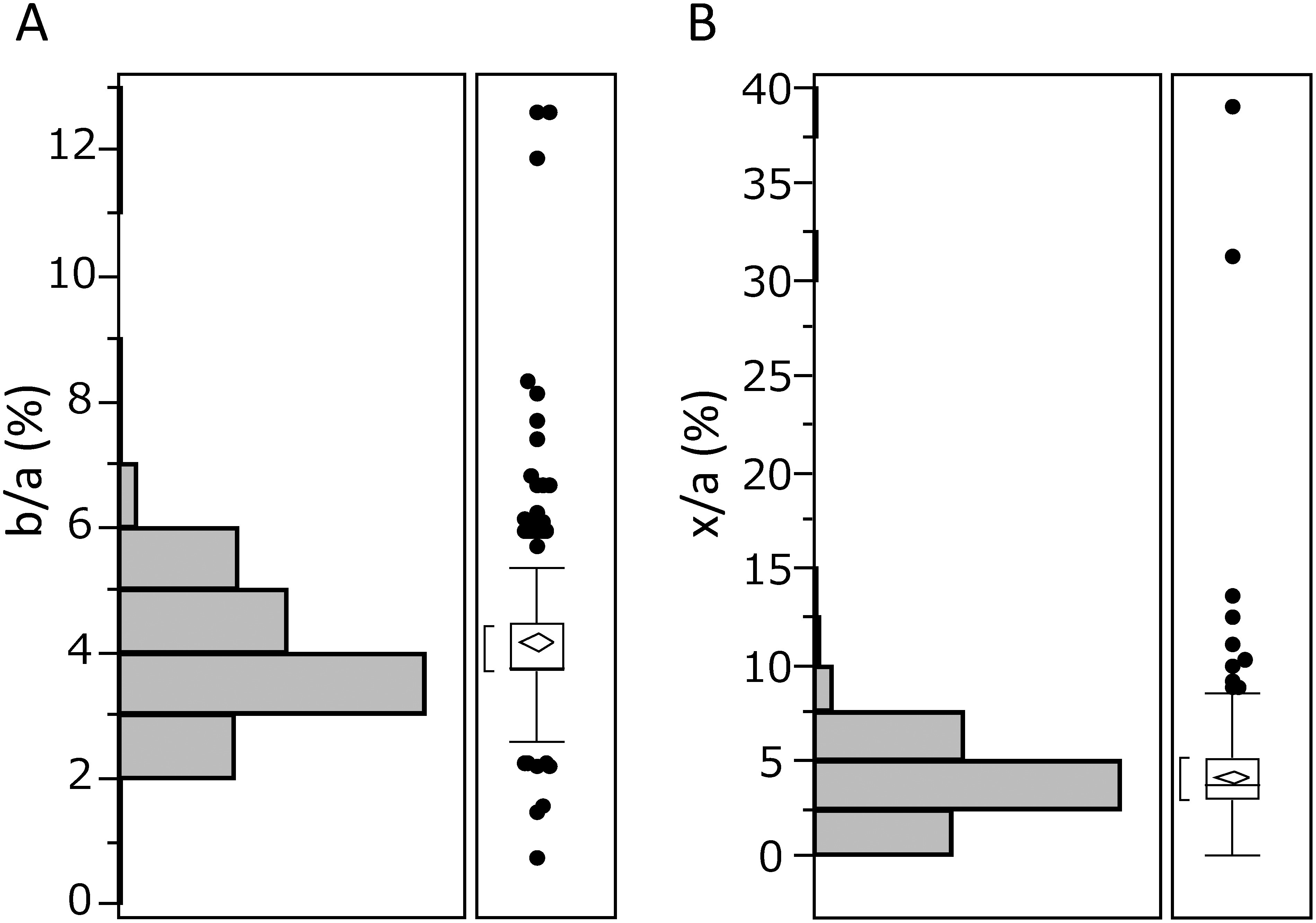
Fig. 9. Distribution of peak ratio (*n*=331). The ratios b/a (A) and x/a (B) indicate the levels of decreased site occupancy and decreased levels of sialylation, respectively. The upper limits of the reference range calculated using 90% sample quantiles was 5.3% and 6.7% for A and B, respectively.

As shown in Fig. 8, sample from the control subjects also showed small peaks corresponding to “b” and “x,” while peaks “y” and “z” were not observed, indicating that a statistical evaluation would be needed to define or diagnose “abnormal.” To this end, the ratio of the intensity of these peaks to peak “a” at *m*/*z* 1,989.9 was calculated in a cohort of undiagnosed 331 samples (Fig. 9). The interquartile range of the b/a ratio was 3.7–4.5% and the upper limit of the reference range was 5.3% when calculated using 90% sample quantiles, and that of the x/a ratio was 3.0–5.2% and the upper limit was 6.7%. The increased ratios of b/a and x/a suggest CDG-I and II, respectively, and patients whose value exceeds the upper limit would be candidates for genetic analysis. For reference, the data for the genetically diagnosed CDG patients are presented in Table 1. The form “b” in PMM2- and ALG14-CDG and the form “x” in SLC35A1- and SLC35A2-CDG were significantly increased. Increases in both “b” and “x” in PGM1-CDG indicate a mixed CDG-I/II type. The deconvoluted spectra were helpful for verifying the presence of these key forms (Supplementary Figure S3).

**Table 1:** Table 1. The b/a and x/a ratios (%) as markers of site-occupancy and sialylation, respectively, of genetically diagnosed patients.

CDG type	b/a	x/a
PMM2	123.0*	0
ALG14	8.8*	1.5
SLC35A1	2.3	21.1*
SLC35A2	1.5	16.2*
PGM1	27.3*	7.8*
MAN1B1	0	0

The calculation is based on the ESI-Q mass spectrum in Fig. 7. *significantly increased.

## CONCLUSION

The molecular mass of transferrin cannot be determined accurately due to the wide distribution of isotope clusters. Molecular phenotyping of CDG can also be achieved in the low *m*/*z* part of the multiply-charged ion mass spectrum. The aberrant molecules that are characteristic of various types of CDG were identified in the *m*/*z* 1,970–2,000 range of the ESI-Q mass spectrum. Even in control subjects, there were small peaks indicative of glycosylation failure or reduced sialylation, so it is necessary to set a cutoff level in order to achieve a valid evaluation.

## Notes

Mass Spectrom (Tokyo) 2022; 11(1): A0103

## References

[1] M. P. Wilson, G. Matthijs. The evolving genetic landscape of congenital disorders of glycosylation. *Biochim. Biophys. Acta, Gen. Subj.* 1865: 129976, 2021. 34358634 10.1016/j.bbagen.2021.129976

[2] P. Lipiński, A. Tylki-Szymańska. Congenital disorders of glycosylation: What clinicians need to know? *Front. Pediatr.* 9: 715151, 2021. 34540767 10.3389/fped.2021.715151 PMC8446601

[3] Y. Wada, A. Nishikawa, N. Okamoto, K. Inui, H. Tsukamoto, S. Okada, N. Taniguchi. Structure of serum transferrin in carbohydrate-deficient glycoprotein syndrome. *Biochem. Biophys. Res. Commun.* 189: 832–836, 1992. 1472054 10.1016/0006-291x(92)92278-6

[4] Y. Wada, P. Azadi, C. E. Costello, A. Dell, R. A. Dwek, H. Geyer, R. Geyer, K. Kakehi, N. G. Karlsson, K. Kato, N. Kawasaki, K. H. Khoo, S. Kim, A. Kondo, E. Lattova, Y. Mechref, E. Miyoshi, K. Nakamura, H. Narimatsu, M. V. Novotny, N. H. Packer, H. Perreault, J. Peter-Katalinic, G. Pohlentz, V. N. Reinhold, P. M. Rudd, A. Suzuki, N. Taniguchi. Comparison of the methods for profiling glycoprotein glycans—HUPO Human Disease Glycomics/Proteome Initiative multi-institutional study. *Glycobiology* 17: 411–422, 2007. 17223647 10.1093/glycob/cwl086

[5] N. Abu Bakar, D. J. Lefeber, M. van Scherpenzeel. Clinical glycomics for the diagnosis of congenital disorders of glycosylation. *J. Inherit. Metab. Dis.* 41: 499–513, 2018. 29497882 10.1007/s10545-018-0144-9 PMC5959975

[6] R. Barone, L. Sturiale, D. Garozzo. Mass spectrometry in the characterization of human genetic *N*-glycosylation defects. *Mass Spectrom. Rev.* 28: 517–542, 2009. 18844296 10.1002/mas.20201

[7] M. van Scherpenzeel, G. Steenbergen, E. Morava, R. A. Wevers, D. J. Lefeber. High-resolution mass spectrometry glycoprofiling of intact transferrin for diagnosis and subtype identification in the congenital disorders of glycosylation. *Transl. Res.* 166: 639–649, 2015. 26307094 10.1016/j.trsl.2015.07.005

[8] B. Casetta, S. Malvagia, S. Funghini, D. Martinelli, C. Dionisi-Vici, R. Barone, A. Fiumara, M. A. Donati, R. Guerrini, G. la Marca. A new strategy implementing mass spectrometry in the diagnosis of congenital disorders of *N*-glycosylation (CDG). *Clin. Chem. Lab. Med.* 59: 165–171, 2021. 10.1515/cclm-2020-0650 32776892

[9] K. Yamashita, T. Ohkura, H. Ideo, K. Ohno, M. Kanai. Electrospray ionization-mass spectrometric analysis of serum transferrin isoforms in patients with carbohydrate-deficient glycoprotein syndrome. *J. Biochem.* 114: 766–769, 1993. 8138529 10.1093/oxfordjournals.jbchem.a124253

[10] Y. Wada. Mass spectrometry of transferrin glycoforms to detect congenital disorders of glycosylation: Site-specific profiles and pitfalls. *Proteomics* 16: 3105–3110, 2016. 27095603 10.1002/pmic.201500551

[11] M. Moini, B. L. Jones, R. M. Rogers, L. Jiang. Sodium trifluoroacetate as a tune/calibration compound for positive- and negative-ion electrospray ionization mass spectrometry in the mass range of 100–4000 Da. *J. Am. Soc. Mass Spectrom.* 9: 977–980, 1998.

[12] M. Tajiri, M. Kadoya, Y. Wada. Dissociation profile of protonated fucosyl glycopeptides and quantitation of fucosylation levels of glycoproteins by mass spectrometry. *J. Proteome Res.* 8: 688–693, 2009. 19099505 10.1021/pr800727w

[13] M. Loos, C. Gerber, F. Corona, J. Hollender, H. Singer. Accelerated isotope fine structure calculation using pruned transition trees. *Anal. Chem.* 87: 5738–5744, 2015. 25929282 10.1021/acs.analchem.5b00941

