# Supplementary material for: Electrospray Ionization Mass Spectrometry of Transferrin: Use of Quadrupole Mass Analyzers for Congenital Disorders of Glycosylation
Source: Mass Spectrom (Tokyo). 2022 Apr 15;11(1):A0103. doi: 10.5702/massspectrometry.A0103 (PMC9395324; doi:10.5702/massspectrometry.A0103)

Supplementry Figure S1
Magnified mass spectrum of PMM2-CDG shown in Figure 5A.
(A) The *m/z* values of $[M+36H]^{36+}$ ions of normal transferrin, $[M+35H]^{35+}$ ions lacking one glycan, and $[M+34H]^{34+}$ ions lacking two glycans were 2210.9, 2211.0 and 2211.1, respectively, and completely overlapped.
(B) The *m/z* value of $[M+33H]^{33+}$ ions lacking one glycan was 2344.9 and that of $[M+34H]^{34+}$ ions of fucosylated normal transferrin was 2345.1, and the corresponding peaks were overlapped.

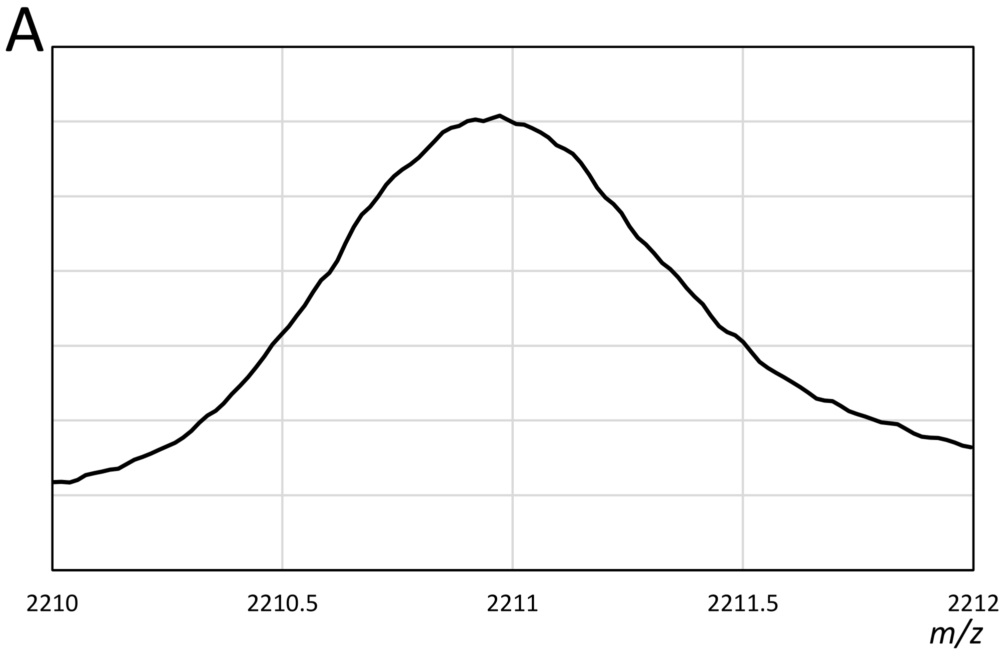

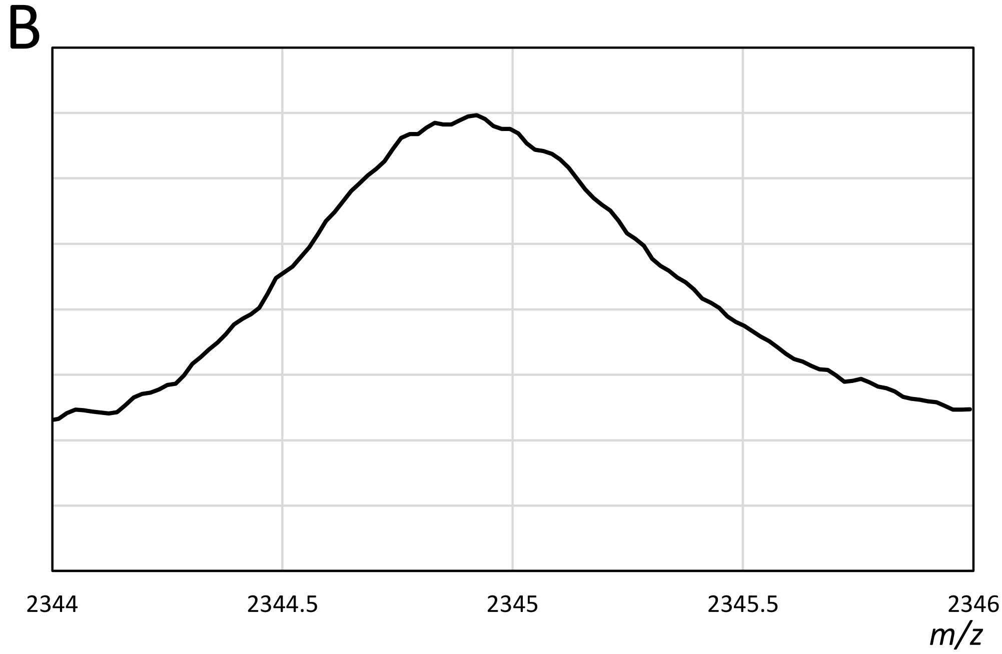

Supplementry Figure S2

Deconvoluted spectra converted with varying parameters. A small diagnostic peak “b” of ALG14-CDG is hardly detected in B and C. Promass decovolution program was used. Minimum peak width is the estimated peak width at peak base of *m/z* peaks in the input ESI mass spectra. Peak intensity cut-off level is a threshold on the intensity relative to the largest peak in the input spectrum.

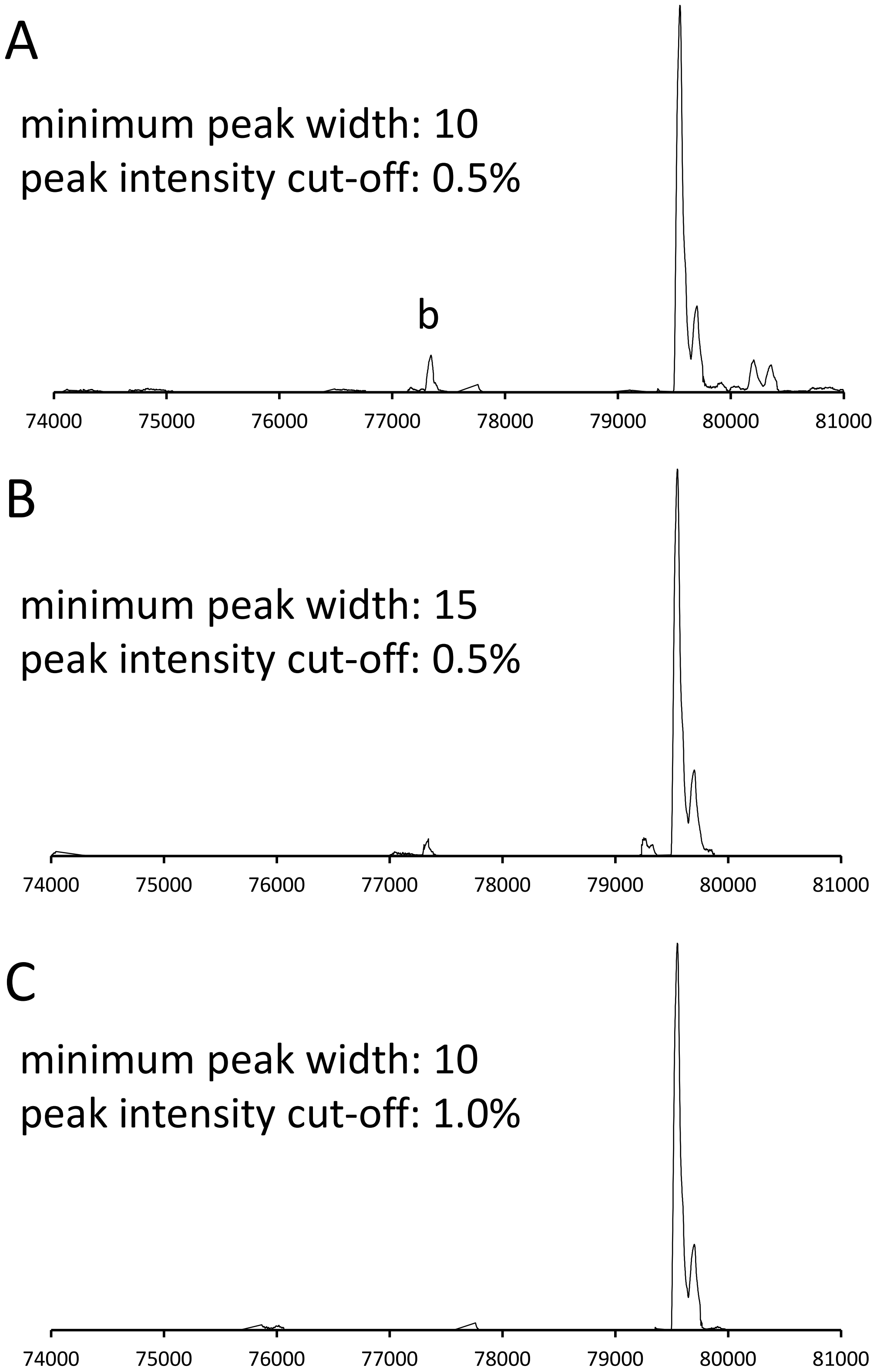

Supplementary Figure S3

Deconvoluted spectra of various types of CDG.

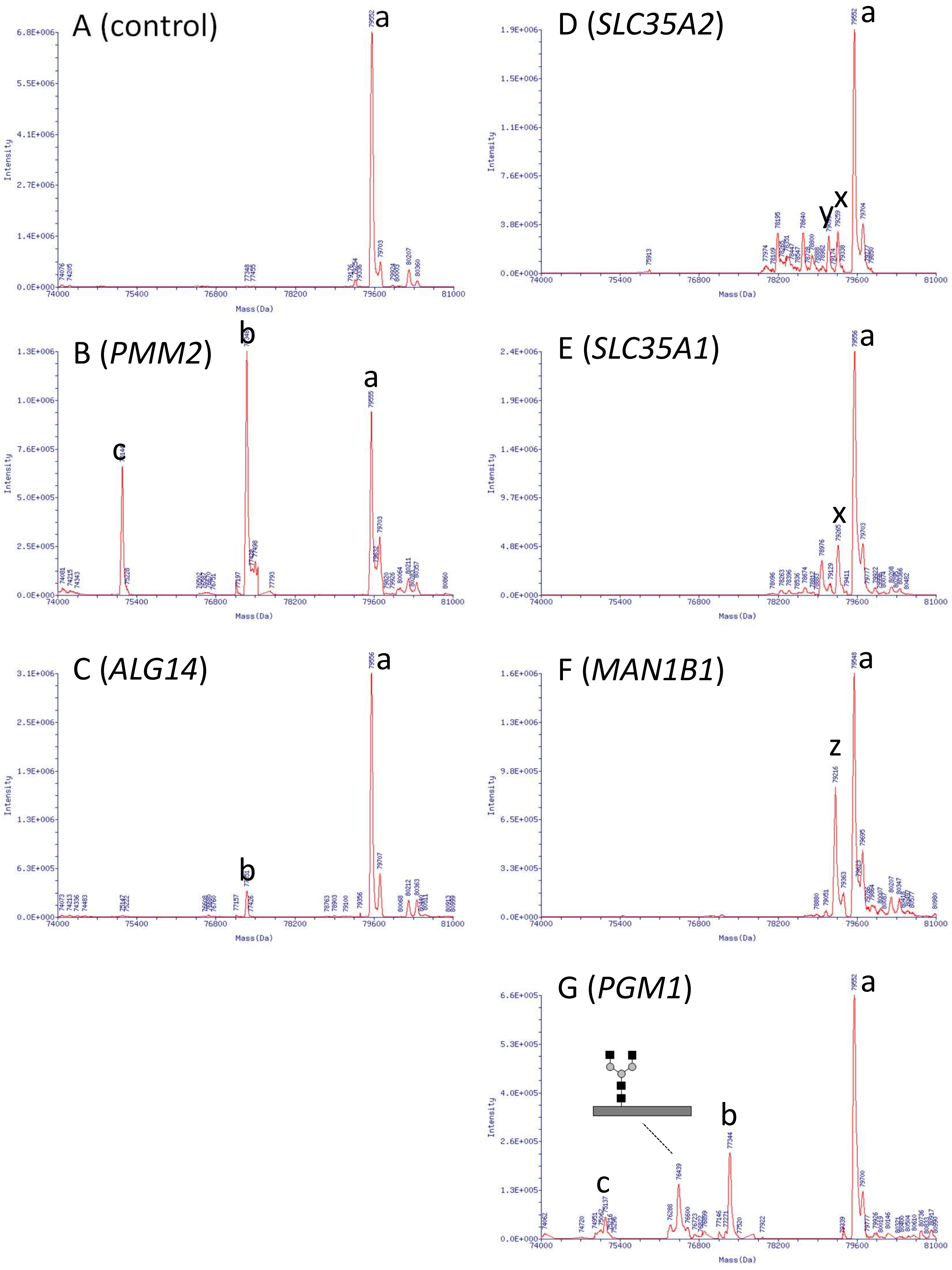

Supplement: Supplementary Data [file massspectrometry-11-1-A0103_s001.pdf]
